# Effect of Fiber Content on the Mechanical Properties of Engineered Cementitious Composites with Recycled Fine Aggregate from Clay Brick

**DOI:** 10.3390/ma14123272

**Published:** 2021-06-13

**Authors:** Zhanqi Cheng, Wenhao Yan, Zhibo Sui, Jiyu Tang, Chengfang Yuan, Liusheng Chu, Hu Feng

**Affiliations:** School of Civil Engineering, Zhengzhou University, Zhengzhou 450001, China; zqcheng@zzu.edu.cn (Z.C.); Y_wh917@163.com (W.Y.); suizhibo1021@163.com (Z.S.); tjy74@zzu.edu.cn (J.T.); chengfang1102@126.com (C.Y.); fenghu@zzu.edu.cn (H.F.)

**Keywords:** recycled fine aggregate (RFA), recycled brick micro-powder (RBM), strain hardening, fiber volume fraction, size effect

## Abstract

In this study, recycled fine aggregate (RFA), also known as recycled brick micro-powder (RBM), was used to completely replace quartz sand for the preparation of green, low-cost ecological engineered cementitious composites (ECO-ECC). RFA was used to replace ultrafine silica sand in the range of 0–100%. Firstly, the optimal replacement rate of RFA was determined, and the test results showed that the ECO-ECC prepared by fully replacing quartz sand with RFA as fine aggregate had strain hardening and multiple cracks, and the tensile strain of the specimens could reach 3%. Then the effects of fiber volume fraction and size effect on the mechanical properties of ECO-ECC were systematically investigated. The results showed that the fiber volume fraction has some influence on the mechanical properties of ECO-ECC. With the increase of fiber volume fraction, the ultimate deflection of the material keeps increasing up to 44.87 mm and the ultimate strain up to 3.46%, with good ductility and toughness. In addition, the compressive strength of the material has a good size effect, and there is a good linear relationship between different specimen sizes and standard sizes. It provides a good basis for engineering applications. Microscopic experimental results also showed that fibers play an important bridging role in the material, and the fiber pull-out and pull-break damage effects are significant.

## 1. Introduction

As a widely used building material, concrete has a serious impact on the ecological environment after it is discarded [1,2]. Nowadays, the world advocates sustainable development. Taking green concrete as the starting point, the use of green materials can promote sustainable development [3,4,5]. It is an important way to reduce construction waste to produce recycled building materials by reprocessing construction waste into aggregate [6].

Engineered cementitious composites (ECC) are a new type of building materials. Li and his co-authors first proposed and developed ECC, and then did many studies to prove the good performance of ECC [7]. Fibers play a very important role in cementitious materials [8]. When the fiber volume fraction of ECC material is less than 2%, it shows strain hardening characteristics under uniaxial tensile test, the maximum strain is greater than 3%, and the crack width developed in the saturated multi-slit cracking state is less than 100 μm [9,10,11,12]. Wang and Li used a size of 304.8 mm × 76.2 mm × 25.4 mm to make ECC specimens and tested the bending performance of specimens with an age of 24 h and 90 days [13]. The test results showed that the bending strength of the ECC specimens reached 11 MPa and 16 MPa at 24 h and 90 days, respectively, and when the age was 90 days, the specimens showed a steady state of multi-slit cracking and stable cracking, and it had better crack control. Yu et al. studied the effect of size on the tensile properties and compressive strength of ECC, and the results showed that in uniaxial tension, the specimens showed strain hardening behavior with multiple micro-cracks; the compressive strength decreases with the increase of specimen size [14]. Liu et al. conducted an experimental study on the permeability of ECC under constant tension load conditions, the test results showed that compared with ordinary concrete, the ECC permeability at the cracking stage was lower than that of ordinary concrete, and its permeability changes with the cubic of local crack width [15]. Marcalikova et al. investigated the effect of straight and hooked fibers on the mechanical properties of fiber-reinforced concrete. The results show that for fiber concrete with straight fibers, typically with higher fiber admixture, the tensile strength in-creases little, but the fracture energy increases significantly; for fiber concrete with hooked fibers, the higher the fiber admixture, the higher the tensile strength and the fracture energy increases significantly, and the bending fibers also have a significant positive effect on the shear resistance [16]. It has been nearly 30 years since the birth of ECC materials. Based on the superior performance of ECC, it has been applied to practical projects in many countries and regions, and has shown good results [17,18].

In recent years, with the continuous development of ECC, on the basis of stability and excellent performance, many scholars have begun to focus on the coordinated development of economy and environment, using recycled aggregates for ECC research, and ECC is developing in a green and economic direction.

Yu et al. studied the tensile and flexural properties of ultra-high performance engineered cementitious composites (UHP-ECC) with different replacement rates of recycled fine powder (RFP) (≤50%) [19]. The results showed that the RFP can promote the hydration of the UHP-ECC matrix and reduce the autogenous shrinkage of UHP-ECC. Zhao et al. used solid waste ceramics powder (CP) as an auxiliary cementing material to replace part of cement to study its uniaxial tensile and bending tests [20]. The results showed that recycling of CP is helpful to clean production of PVA-ECC materials and has good tensile strain and bending toughness. Gao et al. studied the preparation of high ductility cementitious composite (HDCC) from recycled fine aggregates (RFA) [21]. The results showed that HDCC has higher compressive, flexural, bending and tensile strengths. HDCC with RFA has more obvious strain hardening properties, and its bending peak value and the tensile peak value are 182.73% and 183.33% higher than those of HDCC with natural aggregates respectively. Wang et al. studied the effects of different aggregate treatment methods on the properties of recycled aggregates and recycled concrete, the results showed that ‘‘carbonation” significantly improves the mechanical properties and the dry shrinkage resistance of the recycled concrete, however, ‘‘slurry wrapping” greatly enhances the chloride ion penetration resistance, different aggregate treatment methods have different mechanisms to strengthen the interface [22].

In addition, some scholars have conducted research on regenerative ECC from a micro perspective, Thomas et al. studied the use of an innovative technique, computerized microtomography, to evaluate the closed porosity, volume of limestone aggregate fraction and volume of mortar of the multi-recycled aggregate concrete, and the results showed that it is only possible to recycle the concrete a limited number of times [23]. Jalilifar and Sajedi studied the microstructure of 100% recycled concrete with recycled coarse aggregates, after 180 days of hydration, the results showed that the concrete that was not replaced with pozzolanic ash still has a large number of pores and discontinuities in the transition area between the surface and interface of the mortar [24]. Martínez et al. summarized the influence of design parameters on the fresh properties self-compacting concrete with recycled aggregate and commented on the use of recycled aggregate as building materials [25]. Some scholars have also analyzed recycled materials from the perspective of durability [26]. As we know, in the process of urbanization in China, many buildings with masonry structures have been demolished, resulting in a large amount of construction waste mainly made up of clay bricks. At present, to the best of authors’ knowledge, for ECC there is more literature on the use of hollow glass-based, recycled rubber, recycled concrete powder instead of cementitious materials or quartz sand, but there are no experiments on the use of recycled brick micro-powder(RBM) instead of quartz sand to prepare ECC and to investigate the mechanical properties in depth.

Whether ECC can exert strain hardening as well as multi-seam cracking characteristics depends mainly on the cracking strength of the matrix and the strength of the fiber bridging stress. Compared with quartz sand, we chose the waste clay bricks of montmorillonite to prepare RFA [27]. RFA has lower hardness and elastic modulus, and after replacing part of quartz sand, the strength of ECC matrix is reduced, and the stress transmitted by polyvinyl alcohol fiber (PVA) fibers is more likely to crack the surrounding matrix, and more fine cracks appear, which is beneficial to realize the strain hardening of ECC.

In this paper, we present a method to prepare ECO-ECC using waste clay bricks instead of expensive quartz sand. RFA is obtained by crushing the waste bricks in ball mills and sieving. Through our experiments, we hope to achieve our two expected goals, one is to reduce the use of expensive quartz sand, reduce the cost of preparing ECO-ECC and reduce environmental pollution, and the other is that the ECO-ECC prepared by using RFA can obtain good and excellent performance compared with ordinary ECC.

In this paper, the characteristics of RFA are analyzed in detail and the preparation of the experimental procedure is introduced. Then the problem of RFA substitution rate is systematically studied and the optimal substitution rate of the test matrix is proposed. After that, based on the optimal substitution rate, the effects of fiber content and size effect on the mechanical properties of ECO-ECC are studied, and the role of fibers in the matrix material is analyzed from the microstructure. Considering the fiber volume fraction, the peak strength prediction model of ECO-ECC is proposed to provide a basis for the preliminary design and future application of ECO-ECC.

## 2. Experimental Program

### 2.1. Materials and Mix Proportions

**Figure 1:**
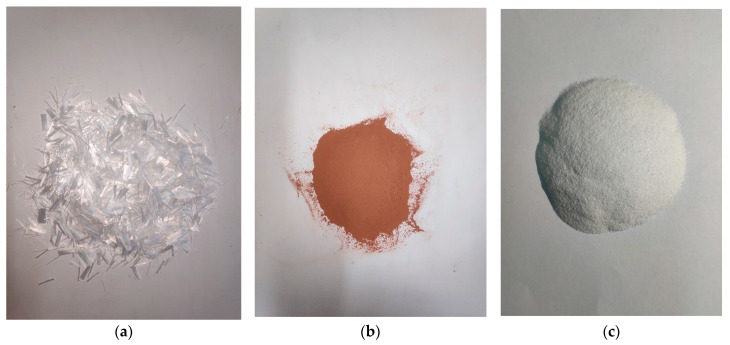
(**a**) PVA fibers, (**b**) RFA, (**c**) quartz sand.

The raw materials in ECO-ECC were ordinary Portland cement (OPC) 42.5, fly ash (FA), quartz sand, RFA and PVA fiber. P.O 42.5 ordinary silicate cement produced by Xingyang City Cement Plant in Henan Province (China) was used for this test. FA was the test grade FA (Grade I) produced by Henan Province Gongyi City Yulian Power Plant. Polyvinyl alcohol fiber (PVA fiber) produced by Kuraray (Shanghai, China) was used. RFA comes from abandoned sintered bricks demolished during construction at Zhengzhou town. The chemical compositions of cementitious materials (OPC, FA) and mechanical properties are presented in Table 1. RFA, quartz sand and PVA fibers are shown in Figure 1. The fine aggregates used in the ECC matrix were quartz sand with the maximum grain size of 300 μm and the mean size of 150 μm to reduce the influence of the weaker interface zone on the properties of ECC materials. The particle size distribution of quartz sand is shown in Table 2. In this study, the PVA fiber with diameter of 40 μm and length of 12 mm, and the aspect ratio of 300, is used for ECC. The main technical indexes of PVA fiber are shown in Table 3.

**Figure 2:**
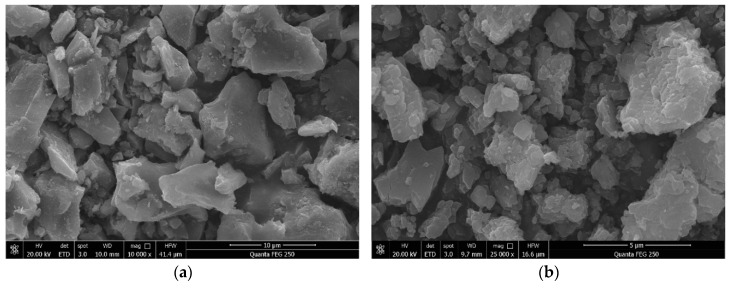
SEM (**a**) RFA, (**b**) quartz sand.

**Figure 3:**
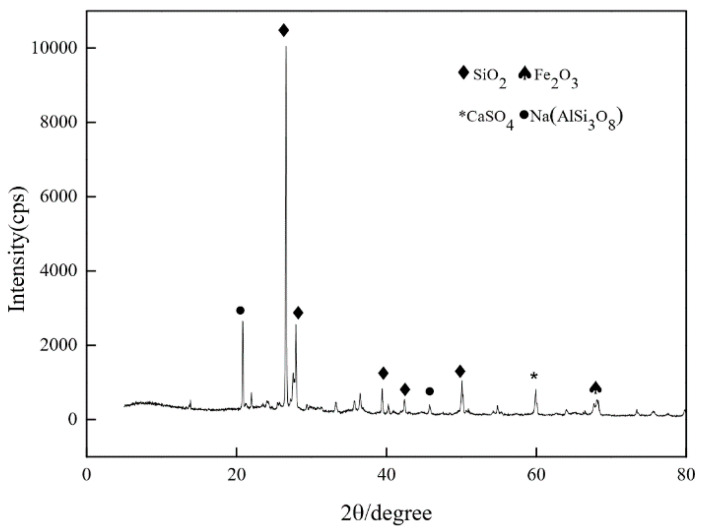
X-ray diffraction (XRD) patterns of RFA.

Figure 2 shows the scanning electron microscope (SEM) (Van-research Intelligence Technology Co., Ltd., Zhengzhou, China) diagram of quartz sand and RFA. It can be seen that the surface of RFA is smoother than quartz sand after crushing and grinding, which can play a better filling role in the reaction mixtures. Figure 3 shows the X-ray diffraction (XRD) (Van-research Intelligence Technology Co., Ltd., Zhengzhou, China) pattern of RFA. RFA is mainly composed of SiO_2_ and Fe_2_O_3_, and contains a small amount of the crystalline component Na(AlSi_3_O_8_). The main components of RFA are the same as those of quartz sand.

In this study, the RFA was obtained by crushing, screening and ball milling of waste sintered clay bricks collected from demolished houses in Henan Province, China. RFA with particle sizes less than 300 μm was used to replace the quartz sand, and its particle size distribution is similar with that of quartz sand in ECC. Because of the higher water absorption of RFA, a polyhydroxy acid superplasticizer was used to ensure the workability. RFA performance indexes are shown in Table 4.

In the present study, two types of mix proportions were prepared. Firstly, in order to study the effects of replacement rate on ECC performance, RFA was used as fine aggregate to partially or completely replace quartz sand in ECC. Five different ECO-ECC mix proportions were prepared, as shown in Table 5. For the reference ECC mixture, ECC-QS, quartz sand is used as fine aggregate. In the ECO-ECC mixtures, ECC-RFA25, ECC-RFA50, ECC-RFA75, and ECC-RFA25 represent that 25%, 50%, 75%, and 100% of quartz sand in the reference mixture were replaced by RFA, respectively. For the other type of mix proportions, when quartz sand in the reference mixture were 100% replaced by RFA, four different ECO-ECC mix proportions were prepared to study the effects of PVA fiber content on the mechanical properties of ECO-ECC, as presented in Table 6. In ECO-ECC-1.25%, ECO-ECC-1.50%, ECO-ECC-1.75%, and ECO-ECC-2%, the PVA fiber contents in ECO-ECC were 1.25%, 1.5%, 1.75% and 2%, respectively.

The preparation process of each mixture is as follows: first the barrel and the arm of the mixer are wetted, and the barrel of the mixer turned to make the surplus water flow out. Then the weighed binding material (cement, FA) and fine aggregate (quartz sand, RFA) are mixed for 2 min, after which water mixed with superplasticizer is added and mixed well at low speed for 2 min to form a uniform solution. After that, fibers were manually and slowly added into the mortar to attain a good dispersion. Next, the thickener was added and stirred for 4 min. The fresh mixture is poured into a steel mold twice, first we pour half of the steel mold capacity, thereafter vibrating for 1 min to ensure the material is dense, then we pour in the remainder, and vibrate for 1 min. Finally, the surface of the specimen was leveled with a trowel. The surface of the poured specimen was covered with plastic film, and the mold was removed after 24 h. the specimens were placed in the standard curing box for curing. The curing temperature and humidity were 20 ± 2 °C and 95%, respectively, and the curing time was 28 days.

### 2.2. Mechanical Properties Tests

For each mixture, to obtain the mechanical properties of ECO-ECC with RFA, six cuboids of 160 mm × 40 mm × 40 mm for flexural testing, six cuboids of 40 mm × 40 mm × 40 mm, 50 mm × 50 mm × 50 mm, 70.7 mm × 70.7 mm × 70.7 mm and 100 mm × 100 mm × 100 mm for compressive testing, three flat plate specimens of 320 mm × 100 mm × 10 mm for four-point bending tests, and three rectangular plate specimens of 280 mm × 40 mm × 15 mm for uniaxial tensile testing each mixture were cast.

According to the “Cement Mortar Strength Inspection Method ISO Method”, the flexural and compressive strength of the test piece are measured. Both the compressive test and flexural test were performed by using a hydraulic servo machine (YAW-300C, (Wuxi Construction Instrument Manufacturing, Wuxi, China). By controlling the loading speed at 50 N/s, the flexural test was performed first to obtain the flexural strength of ECO-ECC. For compressive strength test, 10 t electronic universal testing machine was used. During the compressive test, the compressive fixture is utilized while controlling the loading speed to be constant at 2.4 KN/s.

**Figure 4:**
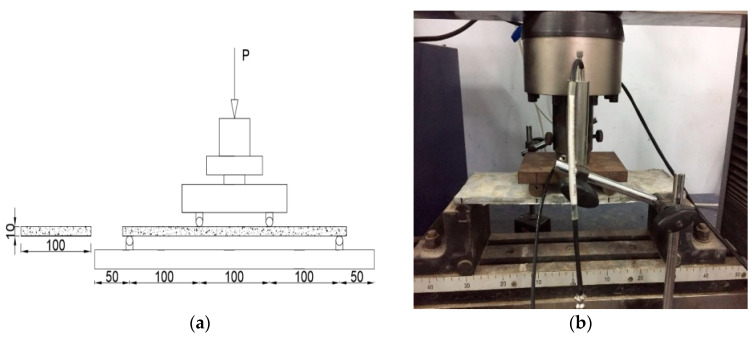
Four-point bending test: (**a**) Schematic representation (dimension in mm); (**b**) Photo of test.

Four-point bending test is an effective method to verify whether the cement-based composite has ultra-high toughness. According to the four-point bending test scheme in Japan Society of Civil Engineers(JSCE) [28], a rectangular flat plate specimen with size of 320 mm × 100 mm × 10 mm was used for the test and the test setup was recommended according to the Chinese Test Standard [29]. As shown in Figure 4a, the 300 mm long equal section area in the middle of the specimen was the deformation and crack observation area. During the test, the displacement control was used, and the loading rate was 0.2 mm/min. The overall trend of the test piece declines or the loading is stopped when the crack width was large. Four-point bending test equipment and bending toughness test were shown in Figure 4b.

**Figure 5:**
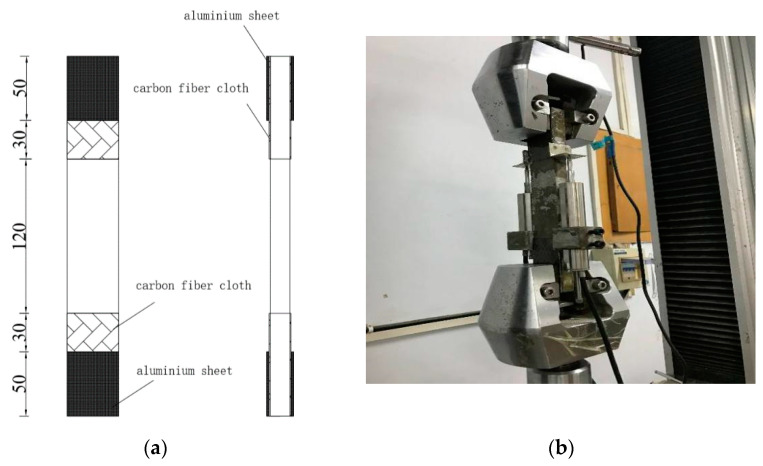
Uniaxial tensile test: (**a**) Schematic representation (dimension in mm); (**b**) Photo of test.

Uniaxial tensile test is an effective method to verify the strain hardening characteristics of cementitious composite materials. Uniaxial tensile tests were conducted on rectangular plate specimens to characterize the tensile behavior of the ECO-ECC mixtures. In this paper, the test was carried out with the rectangular plate specimen with the size of 280 mm × 40 mm × 15 mm. As shown in Figure 5a, the 120 mm equal section area in the middle of the test piece is the deformation and crack observation area, and 80 mm carbon fiber cloth is pasted at both ends to prevent stress concentration. Tests were conducted under displacement control with a loading rate of 0.1 mm/min as recommended by the standard of JSCE [28]. The device for uniaxial tensile test is shown in Figure 5b.

## 3. Results and Discussion

### 3.1. The Effect of RFA Replacement

RFA has been verified to be used to prepare ECC with satisfactory mechanical performance [30,31,32,33,34]. In this sub-section, the influences of the replacement rate of RFA are conducted under the premise of ensuring better performance.

#### 3.1.1. Compressive Strength and Flexural Strength

**Figure 6:**
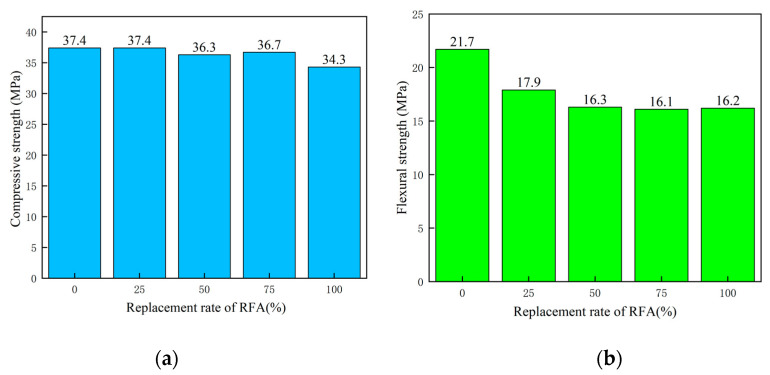
The effects of replacement of RFA in ECO-ECC on compressive strength and flexural strength: (**a**) Compressive strength; (**b**) Flexural strength.

The effects of the replacement rates of RFA on the compressive strength and flexural strength of ECO-ECC with RFA content of 25%, 50%, 75% and 100%, respectively, at 28 days are shown in Figure 6. The compressive and flexural strengths and their standard deviations are shown in Table 7. The results in Figure 6 are the average value of the flexural strength and compressive strength of three specimens for ECC mixture at each substitution rate. Compared with conventional ECC without RFA, the compressive strength of ECO-ECC with the RFA content of 100% slightly decreased from 37.4 MPa to 34.7 MPa. Compared with the compressive strength of the reference ECC with quartz sand as fine aggregate, the compressive strength of ECO-ECC in which the quartz sand is completely replaced by RFA is reduced by about 10%, basically maintaining the same level. This observation corresponds with that of references [35,36]. Although RFA has the similar finesses as quartz sand, it has lower density, much irregular morphology and lower hardness. The low performance of RFA causes a decrease in the compressive strength of ECO-ECC with the replacement rate increases.

The effects of RFA on the flexural strength of ECO-ECC are shown in Figure 6b, where the flexural strength of ECO-RFA25, ECO-RFA50, ECO-RFA75, ECO-RFA100 is 17.9 MPa, 16.3 MPa, 16.1 MPa and 16.2 MPa, respectively, all of them lower than that of reference ECO-QS. It can be seen that when the RFA content in ECO increases from 50% to 100%, the flexural strength remains stable with little change. This observation corresponds with that in references [32,33]. This is because the surface roughness of RFA will increase the internal pores of the structure, reduce the density. And its own material property is poor, which results in the reduction of flexural strength. When the amount of RFA increases, the interfacial bonding force and the bridging stress between the matrix and the fiber can be increased, and the property degradation is slowed down.

#### 3.1.2. Bending Properties

**Figure 7:**
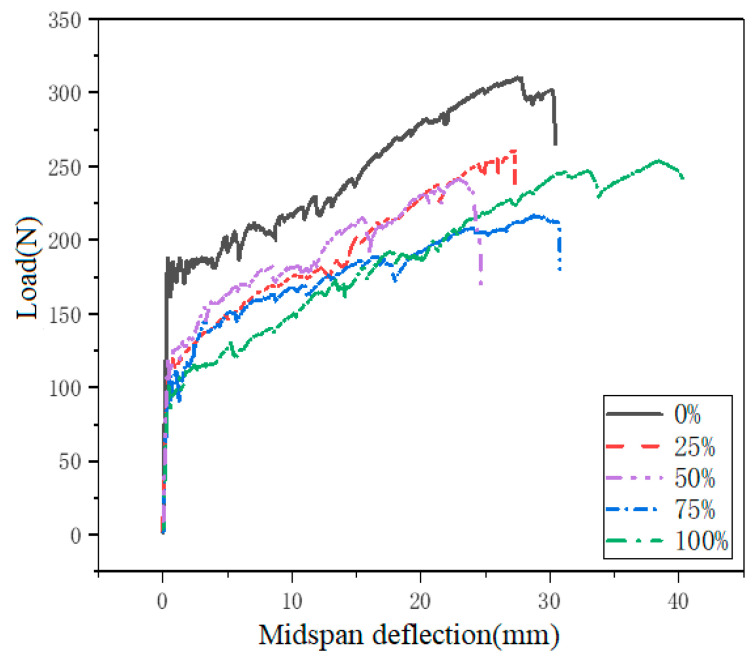
The effects of replacement of RFA on Load-mid-span deflection curves of ECO-ECC under different replacement rates.

The load-mid-span deflection curves of ECC with different RFA contents are shown in Figure 7 and detailed summary of bending parameters is presented in Table 8. At the beginning, all specimens are in the elastic stage. As the external load increases, a single small crack generally tended to open in the constant bending moment region. After cracking, the initial crack continued to propagate to the top surface of the beam, but the width of the crack did not increase significantly, accompanied by multiple tiny cracks parallel to the dominant crack that occurred in the constant moment region. Meanwhile, the strain hardening behavior and multiple cracks along the specimen after initial cracking can be observed. After that, the bridging effect between the fiber, and the cement matrix cannot maintain the tensile stress caused by the external moment, and after reaching the ultimate stress, the bearing capacity of the specimen decreases. In the descending branch of the load-displacement curve, an unstable opening of the main crack was observed. Eventually, the ECC beam specimen failed due to fiber pull-out and significant opening of the main crack in the mid-span. As we all know, the magnitude of the stress fluctuation on the load-deflection curve mainly depends on the strength of the matrix and the bridging ability of the fiber [33].

As listed in Table 8, It can be found that both the first cracking deflection and the ultimate deflection increase with the increase of the replacement rate (25%, 50%, 75% and 100% quartz sand are replaced). On the contrary, for all replacement rates, the first cracking strength and bending peak strength all decrease with the increase of the proportion of RFA in ECO-ECC. Results indicate that the presence of RFA from clay brick does have a profitable effect on the ductility of ECC, while it is not conducive to the strength of the matrix. The lower bending strength was attributed to the lower density, much irregular morphology and lower hardness of RFA.

#### 3.1.3. Tensile Property

**Figure 8:**
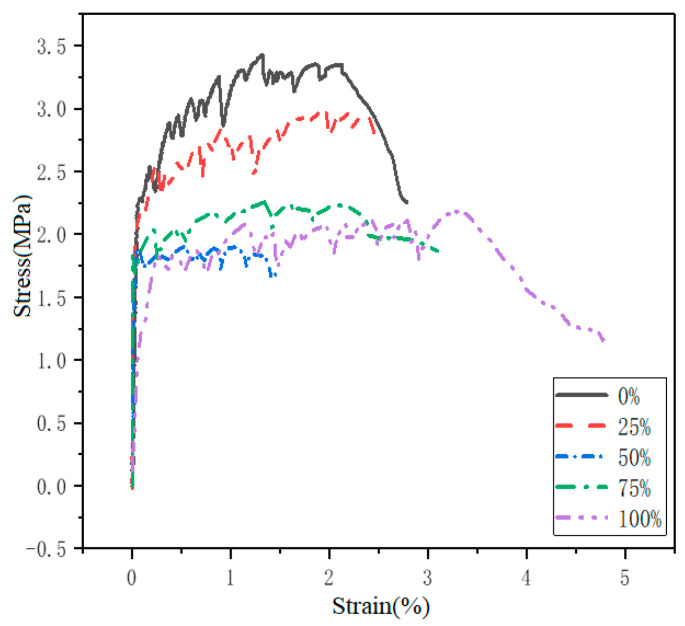
The effect of RFA replacement on uniaxial tensile stress-strain curves of ECO-ECC.

The direct tensile tests were conducted on the flat test specimen to evaluate its tensile properties. The tensile properties are based on the average value of three samples, and the final failure occurs within the gauge length. The uniaxial tensile performance parameters such as the first crack stress *σ_t_*, the first crack strain *ε_t_*, the peak tensile strength *σ_tu_*, the ultimate tensile strain *ε_tu_* can be obtained, as shown in Table 9. The effects of the replacement rates of quartz sand on the stress-strain curve of ECO-ECC at 28 days under uniaxial tension loading are shown in Figure 8.

It can be observed that the ultimate tensile strain of the material is relatively small when the replacement rate of quartz sand with RFA is relatively small (the replacement rate is 25%). However, the ultimate tensile strain of ECO-ECC with the increase of replacement rate, the ultimate tensile strain of ECO-ECC gradually increases, showing good ductility, accompanied by multiple crack and obvious strain hardening phenomenon. When the substitution rate is 100%, the ultimate tensile strain can reach 3.5%, the ductility of the specimen is best compared to other substitution rates. At the same time, it can be found that both the first cracking strength and tensile peak strength decrease with the increase of RFA in ECC, while the tensile strain increases with the increase of RFA in ECC. This observation corresponds with that of Li and Yang [32].

As Yu et al. [33] have reported, the peaking tensile strength of all mixtures was higher than the first cracking stress, indicating the strong strain-hardening and stress harden effect of materials in the post-cracking stage. However, it can also be noticed from Figure 8 and Table 9 that although the increase of RFA from clay brick in ECC is not conducive to the strength of the matrix, it can increase the tensile properties of ECO-ECC, thereby improving its ductility.

After the failure of the specimens, the number of cracks (Nc), the crack space (Sc), and the crack width (Wc) of the test pieces were determined by visual inspection. Nc represents the average number of cracks in the 120 mm gauge measured portion; Sc is the mean crack interval, defined as Sc = 120/Nc mm; Wc is the ratio of the elongation to the number of cracks. The obtained results are demonstrated in Table 10. It is noted that all the five types of specimens exhibit multiple cracking characteristics and that the crack width of microcracks remains rather tight.

**Figure 9:**
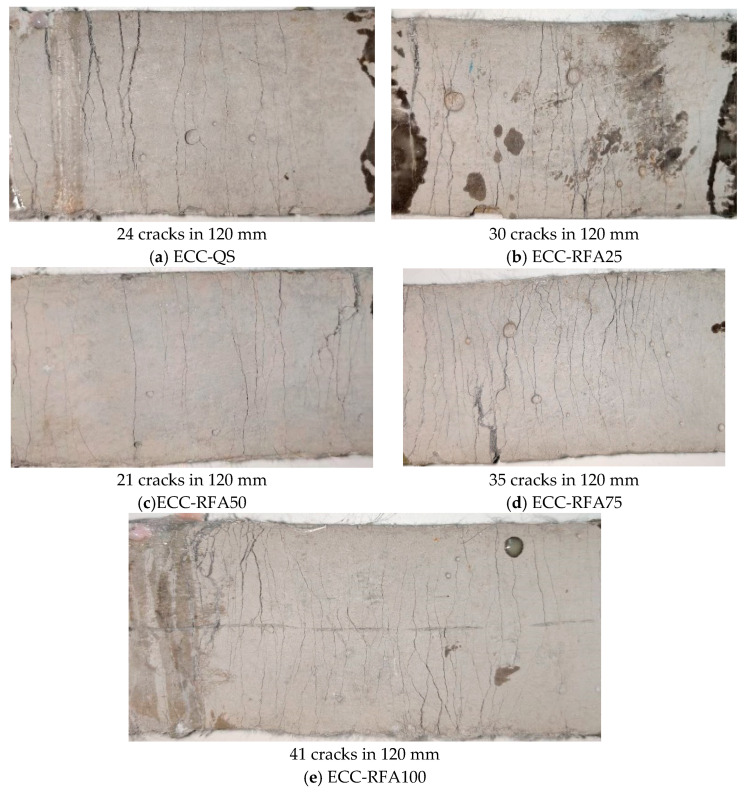
Cracking patterns of the five types of ECC-RFA.

It can be seen from the Figure 9 that when the RFA content is 50%, the crack propagation effect is relatively poor, the number of cracks is relatively small, and the tensile strain is also poor. When the RFA content is 100%, the number of cracks is as high as 41, the average crack spacing is 2.93 mm, and the average crack width is 118 μm. Compared with other groups, when the RFA content is 100%, the ECC-RFA100 performance has the best effect, and the cracks are almost evenly distributed in the entire loading part of the specimen.

According to the above test results, it can be found that when RFA completely replaces quartz sand, the prepared ECO-ECC exhibits a typical strain hardening phenomenon while maintaining excellent compressive performance and flexural performance. In the subsequent studies in this article, the replacement rate of RFA is 100%. For ECC, its ductility depends on the strength of the matrix and the bridging ability of the fiber. In order to better understand the mechanical properties of ECO-ECC, we will discuss the influences of fiber volume fraction on the compressive performance, flexural performance, tensile properties and bending properties of ECO-ECC.

### 3.2. The Effects of PVA Fiber Content

#### 3.2.1. Compressive Properties

The compressive strength of ECO-ECC specimens at 28 days when the FA volume fraction is 35%, the water-binder ratio is 0.35, and the PVA fiber volume fraction is 1.25%, 1.5%, 1.75%, and 2.0% respectively, is discussed in the text that follows.

The damage phenomenon of ECO-ECC is different from that of ordinary concrete. Ordinary concrete cube compressive test block damage can be heard when the test block crumbling sound, and the larger the size of the test piece, the greater the ability of the concrete strength build-up, the greater the brittleness and loudness of the damage, due to the constraints of the loading pad, the damage form for the conical damage surface, this phenomenon reflects the inherent brittleness of the concrete material itself. In order to consider the effect of size effect of ECO-ECC, cubic specimens of 40 mm, 50 mm, 70.7 mm and 100 mm were used for testing in this test, and the results showed that the compression damage process of ECO-ECC was significantly different from that of ordinary concrete, and no conical damage surface appeared in all specimens regardless of the size of the specimens. 

**Figure 10:**
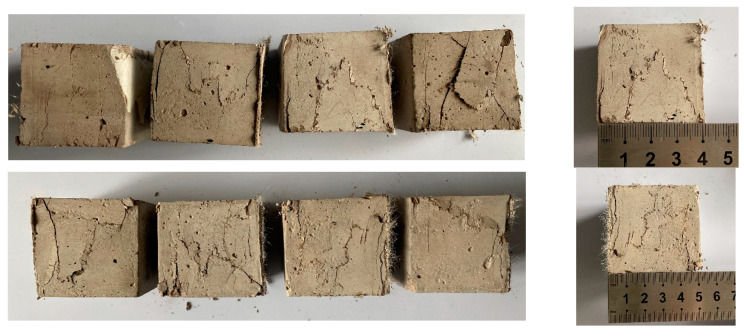

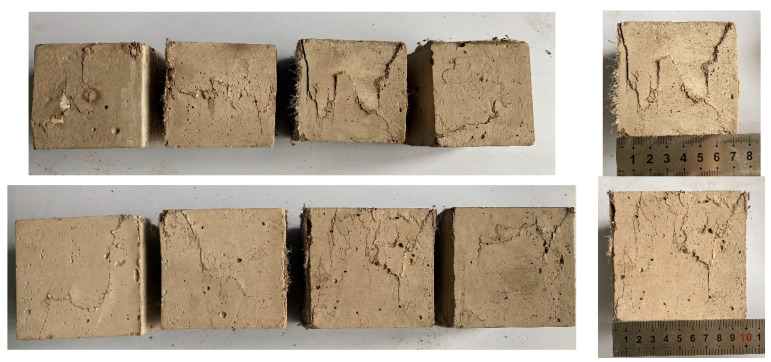
The damage patterns of the four sizes of specimens.

**Figure 11:**
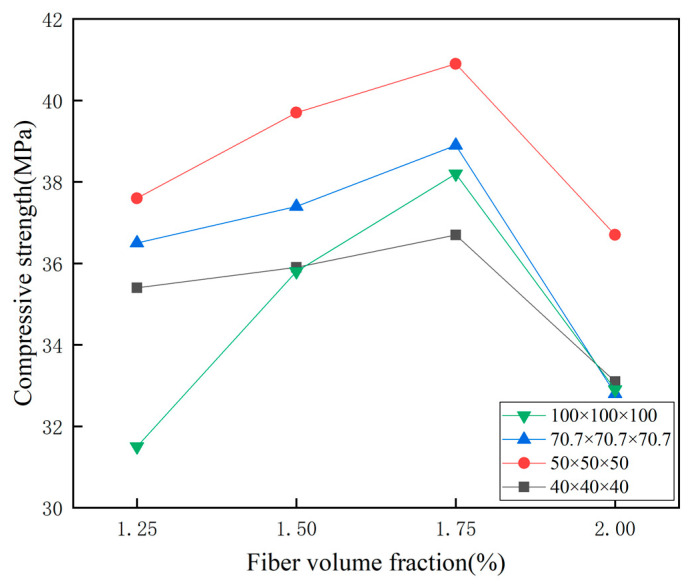
Compressive strength of different sizes with different fibers.

During the compression of the ECO-ECC, the sound of fibers pulling out and pulling off can be heard, and eventually the lateral deformation of the specimen starts to increase significantly, the bearing capacity decreases, and the specimen is damaged. The test phenomena showed that the bridging action of the fibers significantly reduced the brittle properties of the material, which also allowed the material to maintain good integrity after damage. The damage patterns of the four sizes of specimens are shown in Figure 10. The experimentally measured strength of each group of specimens is shown in Table 11 and Figure 11.

As reported by Pan et al. [37], the effect of PVA fiber content on compressive strength involves two opposite aspects. The positive effect is that the compressive strength can be improved by limiting the lateral expansion under load, which helps increase the resistance of the fiber bridge to micro-crack sliding and extension. The negative effect is that as the fiber volume fraction increases, the number of pores will increase and the density will become worse, which will lead to a decrease in strength. The combined influence of fibers resulted in the decrease of the compressive strength as fiber volume fraction increased.

As shown in Table 11 and Figure 11, the compressive strength tends to increase and then decrease with the increase of fiber volume fraction. The reason may be that when the fiber volume fraction is from 1.25% to 1.75%, the crack expansion of the matrix is inhibited and the compressive strength of the matrix is increased due to the toughening and bridging effect of PVA fibers. As can be seen from Figure 11, when the fiber volume fraction is 2%, the compressive strength of the matrix appears to drop abruptly, probably because the fiber volume fraction is too large, when there are too many fibers, which leads to insufficient cement mortar wrapped around the fibers, and at the same time makes the freshly mixed slurry swell and introduces a large number of air bubbles, which reduces the compressive strength of the ECO-ECC.

To further analyze the effect of size effect, a 100 mm cube specimen was used as a standard to calculate the size effect coefficient of cubic compressive strength: see Table 12.

Based on the average size effect and Equation (1) for fitting, where fcu, 100 is the size effect conversion factor, the results are shown in the following equations:

Fcu = δfcu, 100(1)

Fcu, 40 = 1.024 × fcu, 100(2)

Fcu, 50 = 1.123 × fcu, 100(3)

Fcu, 70.7 = 1.055 × fcu, 100(4)

**Figure 12:**
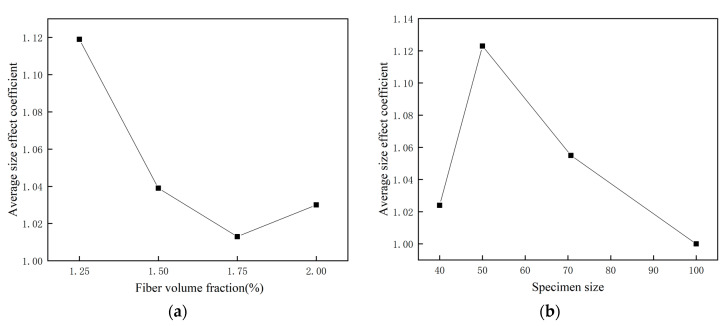
(**a**) the relationship between the fiber volume fraction and the average size effect coeffi-cient; (**b**) the relationship between the specimen size and the average size effect coefficient.

From Equations (2)–(4), it can be seen that there is a size effect between the compressive strength of the 50 mm and 70.7 mm cubes and the 100 mm standard cube specimens. As can be seen from Table 12 and Figure 12b, the compressive strength gradually decreases with increasing cube size for the 50 mm, 70.7 mm and 100 mm cube specimens.

To further analyze the relationship between the size effect and the change of fiber volume fraction, Figure 12a shows the relationship between the fiber volume fraction and the average size effect coefficient, Figure 12b shows the relationship between the specimen size and the average size effect coefficient. It can be seen that the fiber volume fraction has a greater influence on the size effect coefficient, and the decreasing trend of the size effect coefficient is larger with the increase of fiber volume fraction.

#### 3.2.2. Bending Properties

**Figure 13:**
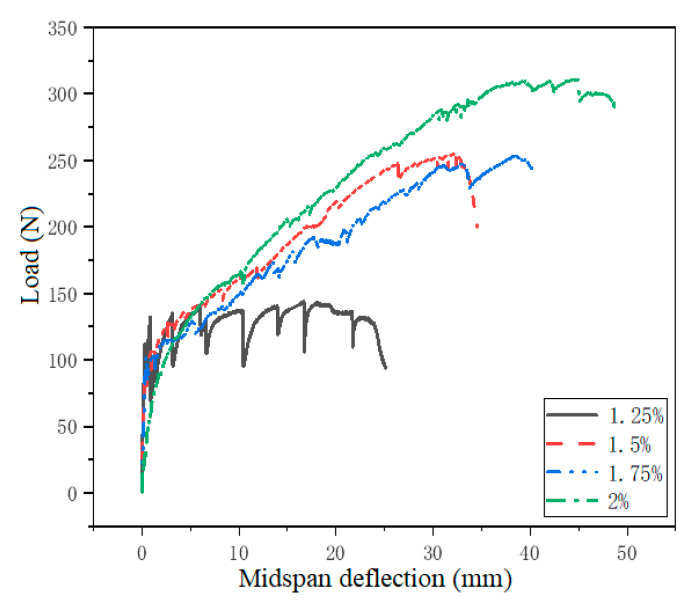
Load-mid-span deflection curves of ECO-ECC with different fiber volume fractions.

The effect of the PVA volume fraction on the recorded bending load-deflection curves, the first cracking strength, the bending peak strength and the tensile strain of ECO-ECC are presented in Figure 13 and Table 13, respectively. All the specimens exhibit multiple cracking with many sub-parallel cracks, and the strain hardening is obvious. As for the effect of PVA fiber volume fraction on the tensile properties, it is clear that PVA fiber volume fraction has significant effect on the strain-hardening and bending strength. As the fiber volume fraction changes from 1.25% to 1.5%, 1.75%, 2%, the mid-span deflection increases from 20.20 mm to 32.93 mm, 38.49 mm and 44.87 mm, respectively. As we all know, the magnitude of the stress fluctuation on the load-deflection curve mainly depends on the strength of the matrix and the bridging ability of the fiber [34]. The lower fiber volume content results in weaker fiber bridging ability. Therefore, the ability to suppress cracks accompanied by obvious strain hardening behavior is lower.

The strain hardening behavior can be expressed in two states, the first cracking state and the ultimate state. The cracking strength of matrix is a significant factor for strain hardening phenomenon. It can be observed that the first cracking strength and the peak strength decrease monotonically with the increase of the PVA fiber volume fraction, while the bending peak strength increase dramatically with the increase of the PVA fiber volume fraction. According to the test results, the bending strength *σ_u_* and fiber volume fraction *V_f_* are subjected to correlation regression analysis. The results are shown in Table 14.

**Figure 14:**
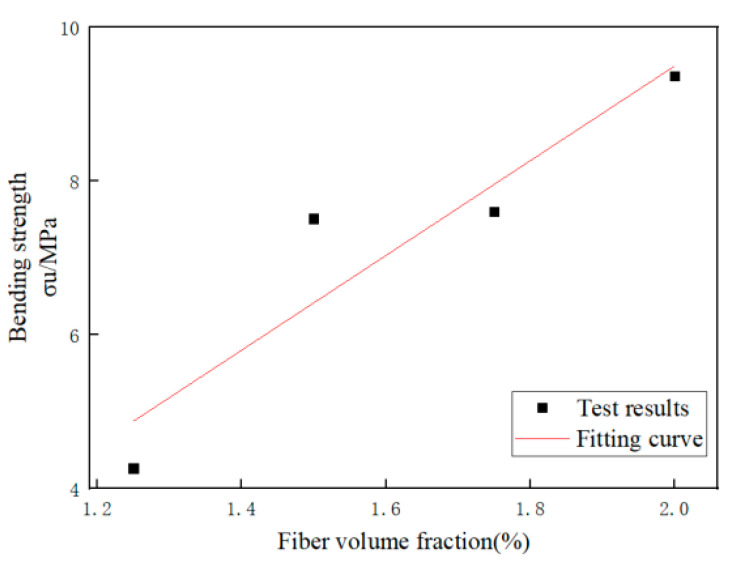
Relationship between bending strength and fiber volume fraction.

It can be seen from Figure 14 that the bending strength of RBP-ECC has a good correlation with fiber volume fraction, and the correlation coefficient is 0.874.

#### 3.2.3. Tensile Properties

**Figure 15:**
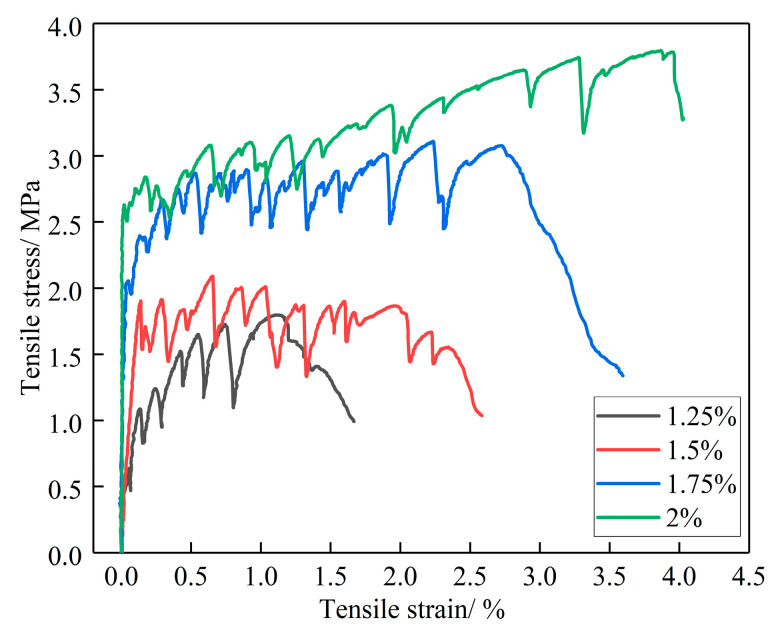
The uniaxial tensile stress-strain curves of ECO-ECC with different fiber volume fractions.

The effect of the PVA volume fraction on the recorded tensile stress-strain curves, the first cracking strength, the tensile peak strength and the tensile strain of ECO-ECC are presented in Figure 15 and Table 15, respectively.

The effect of the PVA volume fraction on the recorded tensile stress-strain curves, the first cracking strength, the tensile peak strength and the tensile strain of ECO-ECC are presented in Figure 15 and Table 15, respectively. All the specimens exhibit multiple cracking with many sub-parallel cracks, and the strain hardening is obvious. As Wang et al. [34] reported, it is clear that fiber volume fraction has significant effect on the strain-hardening and tensile strength. As the fiber volume fraction changes from 1.25% to 1.5%, 1.75%, 2%, the tensile strain capacity increases from 1.31% to 2.04%, 3.00% and 4.19%, respectively. As we all know, the magnitude of the stress fluctuation on the stress-strain curve mainly depends on the strength of the matrix and the bridging ability of the fiber [36]. The lower fiber volume content results in weaker fiber bridging ability. Therefore, the ability to suppress cracks accompanied by obvious strain hardening behavior is lower.

The strain hardening behavior can be expressed in two states, the first cracking state and the ultimate state. The cracking strength of matrix is a significant factor for strain hardening phenomenon. It can be observed that the first cracking strength decreases with the increase of the fiber volume fraction. More interestingly, the influence of fiber content on the peak strength is more complex. When the fiber volume fraction increases from 1.25% to 1.5%, the peak strength firstly increases. However, when the fiber content changes from 1.5% to 2%, the final strength changes little or even slightly decreases. This result is consistent with that of Wang et al. [34]

### 3.3. Microstructural Properties

In order to better analyze the role of fiber in the matrix, tensile specimens of RFA-ECC with fiber volume fraction of 0 and fiber volume fraction of 1.75% were subjected to scanning electron microscopy (SEM) analysis.

**Figure 16:**
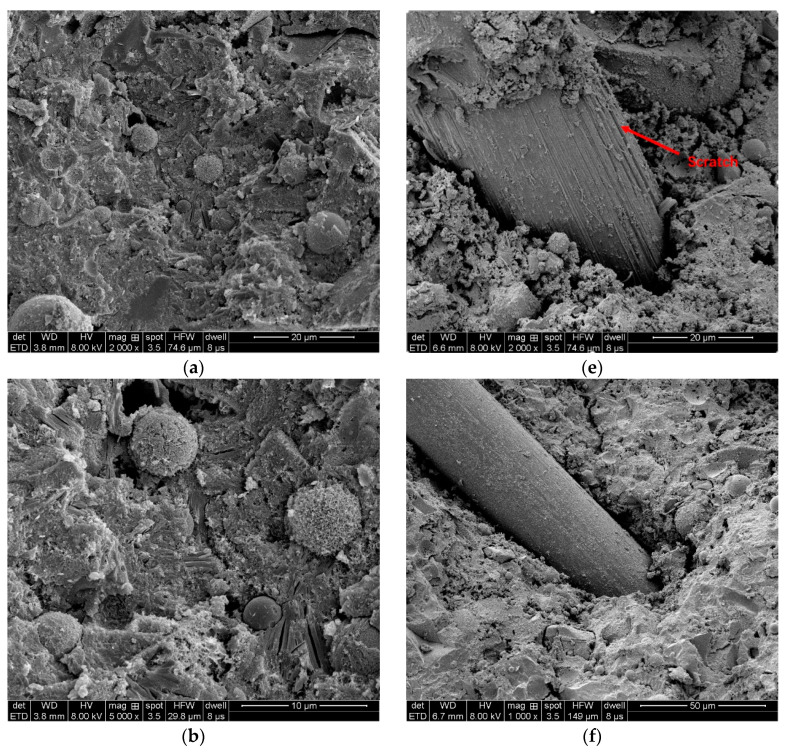

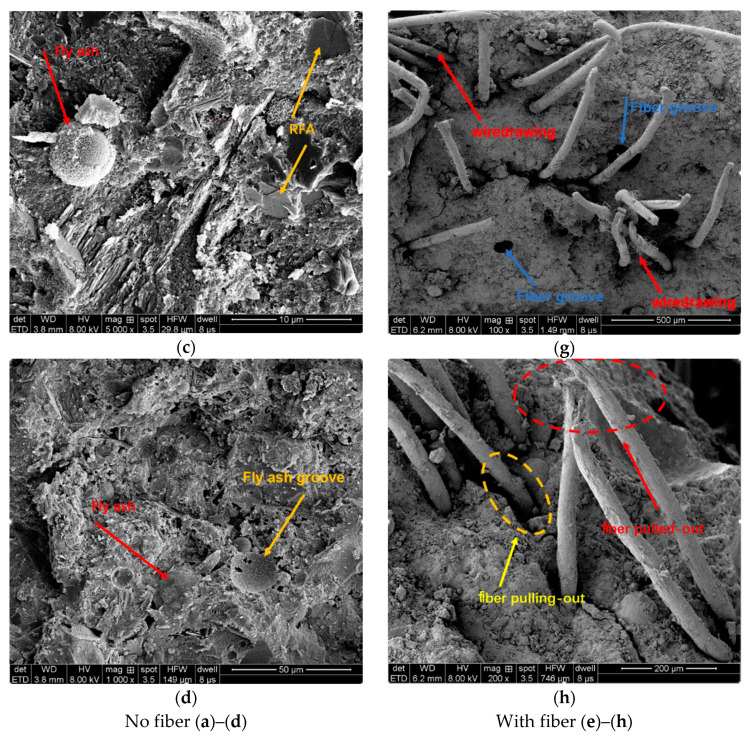
Scanning electron microscopy (SEM).

Figure 16a–d show the SEM with fiber volume fraction of 0. Figure 16a shows the general appearance of the fracture surface structure, and Figure 16b is a partial enlarged figure of Figure 16a, which shows that the fly ash, in the material matrix, plays a good filling role, and the parts of the matrix are denser with each other. Figure 16c shows the incompletely reacted fly ash and RFA, and the RFA is completely embedded in the matrix, tightly bonded to the matrix, and plays an important role in the strength of the material. Figure 16d shows the local fracture surface, which can be seen that the matrix hydration reaction is more complete.

Figure 16e–h show the SEM of fiber volume fraction of 1.75%. Figure 16e shows that there are scratches on the fiber surface due to the frictional bonding between the fiber and the matrix, which strains the fiber itself. Figure 16f shows that the fiber surface is smoother and has less hydration products attached, and the matrix at the root of the fiber is looser, which is a clear characteristic of fiber pullout. Figure 16g shows that some of the fibers have stretched surface and some areas have obvious holes left by fiber slip pulling out, and Figure 16h shows that some fibers break during the pulling out process. In summary, it can be seen that the fiber damage in the matrix material is in the form of fiber pull-out and fiber pull-break.

### 3.4. Shrinkage and Creep

Shrinkage and creep are also factors that discriminate the performance of ECC. According to the available literature, as the recycled brick aggregate increases, the shrinkage of the material increases and the modulus of elasticity decreases [38,39]. In addition, the shrinkage of the material is also related to time; when the recycled brick aggregate replacement rate is low, the shrinkage tends to be stable with time; when the recycled brick aggregate replacement rate is high, the shrinkage increases slowly with time. This is because a large amount of water will be stored in the pores of the recycled brick aggregate to continue the reaction [40]. Compared with the shrinkage value, the creep value does not change that much. Even if the recycled brick aggregate replacement rate is even greater, the creep rate does not exceed 40% [41,42]. For RFA-ECC, its shrinkage and creep follow the concrete law.

## 4. Conclusions

In this study, ECO-ECC was prepared by using RBM with original particle size distribution instead of quartz sand. Cementitious composites were prepared from an eco-friendly perspective, which not only realized the reuse of waste resources, but also made an exploration of eco-friendly material research. The mechanical properties and interfacial microstructure of ECO-ECC prepared by RBM were studied and compared, and the following conclusions were drawn:

(1) The fineness of RFA is similar to that of quartz sand, but the density is lower com-pared to quartz sand, and the surface of RFA is rough, resulting in lower RFA performance. Therefore, the compressive strength of ECO-ECC decreases with the increase of RFA replacement rate.
(2) In the bending test, with the increase of RFA substitution rate, the cracking de-flection and ultimate deflection of ECO-ECC increased, and on the contrary, its cracking strength and ultimate strength decreased. It indicates that RFA has a certain effect on the ductility of ECC, but is not beneficial to the material matrix strength.
(3) With the increase of fiber volume fraction, the compressive strength increases and then decreases, because the increase of fiber limits the transverse cracking of the matrix. Moreover, there is a good size effect between different sizes of compressive specimen.
(4) With the increase of fiber volume fraction, the specimens all showed multi cracking and strain hardening. There is a good linear relationship between fiber volume fraction Vf and bending strength σu, and a linear equation is established.
(5) From the SEM images, it can be seen that the fibers are uniformly distributed in the matrix, and in the damage diagram of the tensile specimen, it can be seen that the fiber damage is mainly tensile and shear damage, and the fiber bridging effect increases the strain of the specimen significantly.

There are some shortcomings in this study. We only made one batch of waste brick powder for the study, and waste clay bricks under different conditions may also have different effects during the experiment. In future research, we will focus on the analytical study of recycled aggregates, and also the deeper investigation of the effect of fiber on the final material properties.

## Figures and Tables

**Table 1:** Chemical component of cementitious materials.

Chemical	OPC	FA	OPC	
Composition (%)			Properties	
Fe_2_O_3_	3.90	4.16	Specific gravity (g/cm^3^)	3.08 ± 0.02
CaO	60.88	4.01	Cement Blaine (m^2^/kg)	370 ± 7.9
MgO	1.75	1.01	Initial setting time (min)	150 ± 9
SiO_2_	21.46	53.97	Final setting time (min)	205 ± 16
Al_2_O_3_	7.28	31.15	Compressive strength (MPa)	-
SO_3_	2.58	-	3 days	25.2 ± 1.9
Na_2_O	-	0.89	28 days	48.5 ± 2.1

**Table 2:** Quartz sand particle size distribution.

Particle Size (μm)	<75	75–125	125–150	150–200	200–300
content (%)	4.25	20.67	34.92	39.72	0.44

**Table 3:** Technical index of PVA fiber.

Diameter (µm)	Length (mm)	Tensile Strength (MPa)	Elastic Modulus (GPa)	Elongation at Break (%)	Density (g·cm^−3^)
40	12	1560	41	6.5	1.3

**Table 4:** Performance indexes of RFA.

Aggregate Type	Apparent Density/kg·m^−3^	Bulk Density/kg·m^−3^	Water Absorption/%	Porosity/%
RFA	2362	993.8	31.8	15.1

**Table 5:** ECO-ECC mix proportion under different replacement rate (kg/m³).

Test Group	Water	Cement	FA	Quartz Sand	RFA	SP	T	PVA Fiber
ECC-QS	415	770	415	415	0	0.6	1.8	22.75
ECC-RFA25	415	770	415	311.25	103.75	0.6	1.8	22.75
ECC-RFA50	415	770	415	207.5	207.5	0.6	1.8	22.75
ECC-RFA75	415	770	415	103.75	311.25	0.6	1.8	22.75
ECC-RFA100	415	770	415	0	415	0.6	1.8	22.75

Note: RFA: recycled fine aggregate; T: thickener; SP: superplasticizer.

**Table 6:** ECO-ECC mix proportion with different fiber volume fractions (kg/m³).

Test Group	Water	Cement	FA	RFA	PVA Fiber	SP	T
ECO-ECC-1.25%	415	770	415	415	16.25	0.6	1.8
ECO-ECC-1.50%	415	770	415	415	19.5	0.6	1.8
ECO-ECC-1.75%	415	770	415	415	22.75	0.6	1.8
ECO-ECC-2%	415	770	415	415	26	0.6	1.8

**Table 7:** Compressive and flexural strength of ECO-ECC with different replacement rates.

Test Group	Compressive Strength (MPa)	Standard Deviation (MPa)	Flexural Strength (MPa)	Standard Deviation (MPa)
ECC-QS	37.4	1.03	21.7	0.39
ECC-RFA25	36.3	0.98	17.9	0.47
ECC-RFA50	36.7	0.78	16.3	0.31
ECC-RFA75	34.3	0.56	16.1	0.29
ECC-RFA100	33.7	0.63	16.2	0.24

**Table 8:** Bending property indexes of ECO-ECC with different replacement rates.

Test Group	First Cracking Deflection *δ_c_*/mm	Ultimate Deflection *δ_c_*/mm	First Cracking Strength *σ_c_*/MPa	Standard Deviation *σ_c_*/(MPa)	Bending Peak Strength *σ_u_*/MPa	Standard Deviation *σ_u_*/(MPa)
ECC-QS	0.366	27.89	5.598	0.34	9.129	0.46
ECC-RFA25	0.695	27.119	3.393	0.24	7.841	0.39
ECC-RFA50	0.368	23.345	3.606	0.29	7.269	0.34
ECC-RFA75	0.532	29.164	3.234	0.27	6.513	0.22
ECC-RFA100	0.976	38.49	3.018	0.20	7.602	0.29

**Table 9:** The effects of replacement of RFA on tensile property indexes of ECO-ECC with different replacement rates.

Test Group	Cracking Strain *ε_ct_* (%)	Cracking Stress *σ_ct_* (MPa)	Standard Deviation *σ_ct_*/(MPa)	Ultimate Strain *ε_ut_* (%)	Ultimate Stress *σ_ut_* (MPa)	Standard Deviation *σ_ut_*/(MPa)
ECC-QS	0.067	2.271	0.22	2.131	3.548	0.31
ECC-RFA25	0.099	2.188	0.16	2.352	2.936	0.34
ECC-RFA50	0.066	1.887	0.18	1.381	1.935	0.29
ECC-RFA75	0.158	1.833	0.13	2.374	2.252	0.25
ECC-RFA100	0.235	1.792	0.20	3.301	2.168	0.21

**Table 10:** Cracking characteristics of each test group.

Test Group	Nc	Wc (μm)	Sc (mm)
ECC-QS	24 ± 3	179 ± 5	5.00 ± 0.71
ECC-RFA25	30 ± 3	151 ± 6	4.00 ± 0.44
ECC-RFA50	21 ± 3	183 ± 7	5.71 ± 0.96
ECC-RFA75	35 ± 5	133 ± 6	3.43 ± 0.57
ECC-RFA100	41 ± 4	118 ± 7	2.93 ± 0.31

**Table 11:** The compressive strength of ECO-ECC with different fiber volume fractions.

Test Group	Fcu, 40	Standard Deviation	Fcu, 50	Standard Deviation	Fcu, 70.7	Standard Deviation	Fcu, 100	Standard Deviation
ECO-ECC-1.25%	35.4	0.679	37.6	0.598	36.5	0.652	31.5	0.438
ECO-ECC-1.50%	35.9	0.579	39.7	0.989	37.4	0.785	35.8	0.964
ECO-ECC-1.75%	36.7	0.435	40.9	0.756	38.9	0.719	38.2	0.856
ECO-ECC-2%	33.1	0.958	36.7	0.245	32.8	0.871	32.9	1.005

**Table 12:** The size effect coefficient of cubic compressive strength.

Test Group	Fcu, 40	Size Effect Coefficient	Fcu, 50	Size Effect Coefficient	Fcu, 70.7	Size Effect Coefficient	Fcu, 100	Size Effect Coefficient	Average
ECO-ECC-1.25%	35.4	1.124	37.6	1.194	36.5	1.159	31.5	1	1.119
ECO-ECC-1.50%	35.9	1.003	39.7	1.109	37.4	1.045	35.8	1	1.039
ECO-ECC-1.75%	36.7	0.961	40.9	1.071	38.9	1.018	38.2	1	1.013
ECO-ECC-2%	33.1	1.006	36.7	1.116	32.8	0.997	32.9	1	1.030
average	-	1.024	-	1.123	-	1.055	-	1	-

**Table 13:** Bending property indexes with different fiber volume fractions.

Test Group	The First Cracking Deflection *δ_c_* (mm)	Ultimate Deflection *δ_u_* (mm)	The First Cracking Strength *σ_c_* (MPa)	Standard Deviation *σ_c_*/(MPa)	Bending Peak Strength *σ_u_* (MPa)	Standard Deviation *σ_u_*/(MPa)
ECO-ECC-1.25%	0.30	20.20	3.36	0.22	4.26	0.19
ECO-ECC-1.5%	0.96	32.93	3.10	0.19	7.51	0.23
ECO-ECC-1.75%	0.98	38.49	3.02	0.16	7.60	0.18
ECO-ECC-2%	1.82	44.87	2.73	0.20	9.36	0.15

**Table 14:** Correlation between bending strength and fiber volume fraction.

Category	Functional Relation	Correlation Coefficient R^2^
*σ_u_* *—* *V_f_*	*σ_u_* = 6.16 *V_f_* − 2.823	0.874

**Table 15:** The tensile property indexes of ECO-ECC with different fiber volume fractions.

Test Group	Cracking Strain *ε_ct_*	Cracking Stress *σ_ct_* (MPa)	Standard Deviation *σ_c_*/(MPa)	Ultimate Strain *ε_ut_*	Ultimate Stress *σ_ut_* (MPa)	Standard Deviation *σ_c_*/(MPa)
ECO-ECC-1.25%	0.189	1.112	0.13	1.31	1.752	0.16
ECO-ECC-1.5%	0.207	1.792	0.16	2.04	2.036	0.14
ECO-ECC-1.75%	0.235	1.892	0.15	3.00	3.368	0.23
ECO-ECC-2%	0.264	2.336	0.18	4.19	3.456	0.21

## Data Availability

The data presented in this study are available on request from the corresponding author.

## Abbreviations

RFA	recycled fine aggregate
RBM	recycled brick micro-powder
ECC	engineered cementitious composites
ECO-ECC	ecological engineered cementitious composites
OPC	ordinary Portland cement
FA	fly ash
PVA fiber	polyvinyl alcohol fiber
XRD	X-ray diffraction
T	thickener
SP	superplasticizer
Nc	number of cracks
Sc	crack space
Wc	crack width
